# Clinicopathological Characteristics of Papillary Thyroid Cancer in Children with Emphasis on Pubertal Status and Association with BRAFV600E Mutation

**DOI:** 10.4274/jcrpe.3873

**Published:** 2017-09-01

**Authors:** Şükran Poyrazoğlu, Rüveyde Bundak, Firdevs Baş, Gülçin Yeğen, Yasemin Şanlı, Feyza Darendeliler

**Affiliations:** 1 İstanbul University Istanbul Faculty of Medicine, Department of Pediatric Endocrinology, İstanbul, Turkey; 2 İstanbul University İstanbul Faculty of Medicine, Department of Pathology, İstanbul, Turkey; 3 İstanbul University İstanbul Faculty of Medicine, Department of Nuclear Medicine, İstanbul, Turkey

**Keywords:** children, pediatric thyroid cancer, papillary thyroid cancer, puberty, BRAF mutation

## Abstract

**Objective:**

Papillary thyroid cancer (PTC) may behave differently in prepubertal children as compared to pubertal children and adults. BRAF gene activating mutations may associate with PTC by creating aberrant activation. We aimed to evaluate the clinicopathological characteristics of PTC patients with emphasis on the pubertal status and also to investigate the association of BRAF^V600E^ mutation with disease characteristics.

**Methods:**

The medical records of 75 patients with PTC were reviewed retrospectively. BRAF^V600E^ mutation status was available only in the medical records of 56 patients.

**Results:**

Mean age at diagnosis was 12.4±3.8 years. There was no difference in sex, initial signs, tumor histopathology, and pathological evidence of tumor aggressiveness between prepubertal and pubertal children. Although not statistically significant, lateral neck nodal metastasis and lung metastasis at diagnosis were more prevalent in prepubertal children. After excluding patients with microcarcinoma, prepubertal children were found to require lateral neck dissection and further doses of radioactive iodine more frequently than pubertal patients. Recurrence was also more frequent in prepubertal children (p=0.016). Frequency of BRAF^V600E^ mutation was similar in prepubertal and pubertal patients. BRAF^V600E^ mutation was found in 14/56 (25%) patients and was high in the classic variant PTC (p=0.004). Multicentricity was high in BRAF^V600E^ mutation (p=0.01). There was no relation between BRAF^V600E^ mutation and lymph node and pulmonary metastasis at diagnosis, or between BRAF^V600E^ mutation and pathological evidence of tumor aggressiveness.

**Conclusion:**

PTC is more disseminated in prepubertal children. BRAF^V600E^ mutation does not correlate with a more extensive or aggressive disease. BRAF^V600E^ mutation is not the cause of the differences in the biological behavior of PTC in prepubertal and pubertal children.

**What is already known on this topic?**

Papillary thyroid cancer (PTC) is more disseminated in prepubertal children. Recurrence rate was reported to be higher in the prepubertal group.

**What this study adds?**

BRAF^V600E^ mutation is not correlated with a more extensive or aggressive disease in pediatric PTC patients. Frequency of BRAFV600E mutation is similar between prepubertal and pubertal children with PTC.

## INTRODUCTION

Differentiated thyroid carcinomas (DTCs) are the most common endocrine neoplasia in childhood. Papillary thyroid cancer (PTC) which arises from follicular epithelial cells constitutes more than 90% of thyroid cancer cases (1). Over the last decades, the incidence of thyroid cancer showed an increase worldwide and the incidence of DTC in children has been reported to increase by 1.1% per year (2).

DTC has been considered a distinct disease in both children and adults. Pediatric DTC differs from the condition in adults in terms of clinical manifestations and outcomes. Lymph node metastasis, extrathyroidal involvement, and pulmonary metastasis at diagnosis are more common in children (3,4,5). Despite extensive disease at diagnosis, children have a more favorable outcome and a lower mortality. Also, transformation to less differentiated tumors is less common in children (4,5,6). Similarly, PTC has been found to behave differently in prepubertal children than in pubertal children. Some studies report an increased prevalence in extrathyroidal involvement, regional lymph node metastases, distant metastases, and lymph node recurrence in younger children (7,8,9,10), but these features are not observed in all studies (11,12). It remains uncertain whether prepubertal children are at greater risk for more extensive disease or for higher rates of recurrence. The recent pediatric guidelines of the American Thyroid Association in 2015 recommend that prepubertal and pubertal status should be included in studies to increase uniformity and to evaluate more accurately the potential influence of pubertal development on the incidence and behavior of DTC within the pediatric population (13).

B-type RAF kinase (BRAF) is a cytoplasmic serine/threonine kinase and an essential molecule in the mitogen-activated protein kinase (MAPK) pathway (14,15). BRAF gene activating mutations cause PTC in thyroid follicle cells by creating aberrant activation and the most common mutation is BRAF^V600E^ (a valine by glycine substitution at codon 600) (14). BRAF^V600E^ mutation may be associated with aggressive character in PTC, reducing differentiation of the cancer and radioactive iodine (RAI) retention capacity by decreased expression of the sodium-iodide symporter (15,16). Although the impact of BRAF^V600E^ mutations in PTC is controversial, some studies in adults showed that BRAF^V600E^ mutation may associate with more aggressive disease, higher risk for lymph node involvement, distant metastasis, and poor prognosis (17,18). It has been suggested that the different behavior of PTC in children and in adults might result from the differences in the prevalence of the BRAF^V600E^ mutation (19,20).

In this study, we aimed to evaluate the clinicopathological characteristics of PTC patients with emphasis on the pubertal status of the patients. We also investigated the association of BRAF^V600E^ mutation with disease characteristics in our patients with PTC.

## METHODS

Between 1983 and 2015, eighty-four patients (56 girls, 28 boys) with TC were followed in our unit. The medical records of these patients were reviewed retrospectively. PTC was the most common type (75 patients, 89.2%), followed by medullary thyroid cancer (MTC) (5 patients, 6%), and follicular thyroid cancer (FTC) (4 patients, 4.8%). MTC and FTC cases were excluded and PTC patients were evaluated in this paper.

The details of the patients’ presentations, family history, pathological findings of the tumor, treatment, and outcome data were evaluated from the medical records of the patients. A history of external radiotherapy to the cervical region prior to admission was questioned in all cases. The dose and frequency of radioiodine (I^131^) ablation therapy and the presence of metastases, recurrences, and other complications in the follow-up were noted. BRAF^V600E^ mutation status was found in the medical records of 56 patients. BRAF^V600E^ mutations of some of the cases were reported previously (21). The patients were classified with respect to pubertal status at diagnosis. The onset of puberty was defined according to Tanner standards as attainment of breast budding in girls and testicular volume ≥4 mL in boys (22).

Treatment for PTC was based on surgery and I^131^ ablation and suppressive thyroid hormone therapy. Sixty-five (86.7%) patients had their thyroid surgery performed at our hospital, whereas the other 10 patients (13.3%) underwent thyroid surgery at other hospitals, and then were referred to our hospital for follow-up and I^131^ therapy. Except the early years when subtotal thyroidectomy was performed; all patients underwent total thyroidectomy with or without neck lymph node dissection. Radioiodine treatment was used for ablation of thyroid remnants and/or lung metastases. All patients except those with microcarcinoma with no risk factors were treated with radioiodine to ablate the postsurgical thyroid remnant. The postoperative radioiodine treatment was prescribed as follows: 30-100 mCi for patients with a tumor limited to the thyroid gland; 150 mCi for tumors invading the thyroid capsule surrounding tissues and/or with metastases in the neck or mediastinal lymph nodes; 175-200 mCi for distant metastases. Dose adjustments in younger children were made on a per kg basis using the doses for a standard 70 kg person as a reference point. Repeated I^131^ treatments were given to patients with evidence of recurrence or metastases. One week after I^131^ administration, a whole-body scintigraphy (WBS) was performed. A second WBS was performed using 2-5 mCi at 12 months after I^131^ treatment concurrent with measurement of stimulated thyroglobulin (Tg) levels before WBS. All patients were maintained on L-thyroxin suppressive therapy.

Patients were followed according to a standard protocol. During follow-up, all patients were evaluated clinically at 3 to 6 month intervals and were given L-thyroxin treatment postoperatively aiming to keep thyroid-stimulating hormone (TSH) levels below 0.1 µU/mL and Tg levels as undetectable. The follow-up protocol included assessment of free thyroxine, TSH, and Tg levels every 3-6 months. Ultrasonography was performed every six months to assess for presence of residual thyroid tissue and evaluation of the lymph nodes. I^131^ WBS was performed using 2-5 mCi 12 months after diagnosis. Concurrent measurements of stimulated Tg levels were also done before WBS and repeated 6-12 months after each radioiodine therapy. Beginning in the 1990s, thyroglobulin levels and thyroglobulin antibodies were monitored in all patients.

Patients were classified as remission, persistent disease, or recurrence. Remission is defined as negative diagnostic results on WBS, neck ultrasound, chest computed tomography, and undetectable or low serum stimulated Tg levels. Patients who never entered remission were accepted as having persistent disease by one or more than one of the following criteria: detectable serum Tg levels either suppressed or after TSH stimulation, lymph nodes on the neck ultrasound, confirmed by fine needle aspiration biopsy and positive WBS. Recurrence was accepted as the appearance of cancer with new RAI uptake or biopsy in any patient who had been free of cancer. If tumor diameter was ≤1 cm, PTC was classified as microcarcinoma.

All of the histological examinations were reviewed at our hospital. Indicators of tumor aggressiveness such as multicentricity, vascular invasion, perineural invasion, thyroid capsular invasion, extrathyroidal extension, lymph node metastasis, and lung metastasis were evaluated.

BRAF^V600E^ mutation analysis was performed in formalin fixed, paraffin-embedded papillary thyroid carcinoma specimens. For genomic DNA preparation, the QIAamp DNA tissue kit (Qiagen, Hilden, Germany) was used following the manufacturer’s instructions. BRAF^V600E^ mutation was determined by pyrosequencing using the Qiagen PyroMark Q24 pyrosequencer (Qiagen, Venlo, Netherlands) following the manufacturer’s instructions, as has been described (23).

Statistical analyses were performed using SPSS statistical package version 17 (SPSS Inc., Chicago, IL, USA). The results are presented as mean ± standard deviation or as percentage figures. Chi-square test, Fisher’s exact test, t-test, and Kruskal-Wallis test were used in the statistical analyses. A p-value <0.05 was accepted as statistically significant.

This study was approved by the local ethics committee.

## RESULTS

Mean age of the patients at diagnosis was 12.4±3.8 years (range 1.3 to 17.8). The male/female ratio was 24/51 (1:2.1) in the total group. The most common presenting sign was presence of a nodule (70%). Twelve patients had a history of radiotherapy for conditions including Hodgkin’s lymphoma (n=7), medulloblastoma, neuroblastoma, pinealoblastoma, pons glioma, and liposarcoma (each in 1 patient). Hodgkin’s lymphoma and PTC were diagnosed simultaneously in one patient. The mean interval between radiotherapy and presentation with PTC was 9.2±2.1 years (range: 6-11 years). PTC was detected in these patients as a result of prospective follow-up based on their history of external radiotherapy. Twenty patients had a family history of thyroid disease. Eighteen patients had an associated disease at diagnosis (13 Hashimoto’s thyroiditis, 2 type 1 diabetes, 2 multinodular goiter, 1 Graves’ disease). Except one, none of the patients had a family history of thyroid cancer.

Total and subtotal thyroidectomies were performed in 92% and 8% of the patients, respectively. Central compartment neck dissection was performed in 42 (56%) patients, lateral neck dissection in 14 (18.7%). Because of multicentricity and history of radiotherapy, 8 patients with microcarcinoma underwent central neck dissection. Neck dissection was performed bilaterally in 7 (9.3%) patients. Temporary hypoparathyroidism was observed in 3 patients (4%) and permanent hypoparathyroidism in 6 (8%) patients after surgery.

The mean tumor diameter was 2.2±1.6 cm (range: 0.2-7 cm) and 23 (30.7%) tumors were microcarcinomas. In 23 microcarcinoma patients, the thyroid nodule was identified incidentally on ultrasound during follow-up.

Twelve patients were followed for history of thyroid disease [Hashimoto thyroiditis (n=6), goiter (n=5), hypothyroidism (n=1)], 8 patients for history of radiotherapy, and 3 patients had thyroid ultrasound because of family history of thyroid disease.

Forty patients (53.3%) had the classical variant of PTC, 29 patients (38.7%) had been diagnosed with the follicular variant, 5 patients (6.7%) with the variant with diffuse sclerosis, and 1 patient (1.3%) with the solid variant of PTC. At diagnosis, the incidence of multicentricity, capsule invasion, lymph node metastasis, and lung metastasis were calculated as 49.3%, 40%, 45.3%, and 13.3%, respectively. In Table 1, some clinical and laboratory features of PTC in prepubertal and pubertal children are presented. Male/female ratio was similar in prepubertal and pubertal children (p=0.56). There was no difference in sex, clinical signs at diagnosis, and tumor histopathology between prepubertal and pubertal children. Presence of BRAF^V600E ^mutation was also similar in prepubertal and pubertal children. Because frequency of microcarcinoma was higher in the pubertal group (36.8%, p=0.04) as compared to the prepubertal group (11.1%), prepubertal children had greater tumor size than pubertal children (p=0.03). However, after excluding microcarcinoma, tumor size was similar between the two groups (Table 2, p=0.24). Similarly, there was no difference in pathological evidence of tumor aggressiveness (multicentricity, vascular invasion, perineural invasion, capsule invasion, extrathyroidal extension) between prepubertal and pubertal children.

Total thyroidectomy and subtotal thyroidectomy ratio was also similar between prepubertal and pubertal children (p=0.62). Radioiodine therapy was administered to 61 patients with PTC in a total dose ranging from 13 mCi to 605 mCi. A total of 17 patients underwent a second or more doses of RAI treatment due to presence of lymph node and lung metastasis. Extent of metastasis, response to treatment, and outcome in patients with clinically detected PTC in prepubertal and pubertal groups are given in Table 2. To be able to compare the two groups, patients with microcarcinoma detected during follow-up for other reasons were excluded. Although lateral neck nodal metastasis and lung metastasis at diagnosis were more frequent in prepubertal children (43.7% and 33.3%, respectively) than in the pubertal group (19.4% and 13.9%, respectively), there was no statistically significant difference in frequency of lymph metastasis and lung metastasis between the two groups after excluding microcarcinoma. Although not statistically significant, a greater proportion of prepubertal children required lateral neck dissection and a second or more doses of RAI treatment for lymph node and lung metastasis compared to pubertal children (Table 2; 43.7% and 19.4% vs. 50% and 25%, respectively). Frequency of persistent disease was similar between prepubertal and pubertal groups. However, recurrence was more frequent in prepubertal children after excluding microcarcinoma patients (25% vs. 2.7%, respectively; p=0.016). Frequency of surgical complications was similar in the prepubertal and pubertal groups. There was no difference with regard to presence of BRAF^V600E^ mutation between prepubertal and pubertal patients after excluding microcarcinoma patients (Table 2, p=0.68).

The mean follow-up period was 4.3±3.4 years (range: 1-14.1). During the follow-up, tumor recurrence was detected in 5 PTC patients. Pulmonary recurrence was observed in 3, cervical lymph node recurrence in 1, and cervical and mediastinal lymph node recurrence in 1 patient. Four of these patients were prepubertal at diagnosis. One patient diagnosed prepubertally died with pulmonary and mediastinal involvement 10.3 years after diagnosis. She had undergone two surgical interventions (total thyroidectomy, neck dissection) and had also received RAI treatment (cumulative dose of 560 mCi).

BRAF^V600E^ mutation was found in 14/56 (25%, 2 prepubertal, 12 pubertal) patients. None of the patients with BRAF^V600E^ mutation had a history of external radiotherapy. The relationship between clinicopathological characteristics of PTC and BRAF^V600E^ mutation is shown in Table 3. While the frequency of BRAF^V600E^ mutation was significantly higher in patients with classical PTC histology (p=0.004), it was similar in girls and boys (p=0.73), and in tumors larger or smaller than 1 cm in diameter (p=0.33). Multicentricity was significantly high in BRAF^V600E^ mutation positive patients (p=0.01), but lymphovascular invasion, perineural invasion, thyroid capsular invasion, and extrathyroidal extension of the tumor were similar between BRAF^V600^E mutation positive and negative patients. There was no relationship between BRAF^V600E^ mutation and lymph node and pulmonary metastasis at diagnosis. Within the pubertal group, there was no difference in tumor aggressiveness with respect to presence of BRAF^V600E^ mutation.

After excluding patients with a history of radiotherapy, when numbers of patients diagnosed per each 10 years during the study period were evaluated, an increase was noted in numbers of cases in the last decade, especially in microcarcinoma (Table 4). The increase, especially in the last decade, of the frequency of PTC in girls and in pubertal patients was noteworthy.

## DISCUSSION

In our cohort, at diagnosis, although not statistically significant, lateral neck nodal metastasis and lung metastases were more frequent in prepubertal children after excluding patients with microcarcinoma. Frequency of microcarcinoma was high in pubertal patients; PTC was also more disseminated in prepubertal children compared to pubertal children. As a result of this, the prepubertal children in our cohort required more lateral neck dissection and more than one dose of RAI therapy than pubertal children. While PTC was more disseminated in the prepubertal group at diagnosis, pathological evidence of tumor aggressiveness was similar between the prepubertal and pubertal groups.

Similar to our results, it was reported that at diagnosis, DTC is generally more widespread at presentation in prepubertal children than in adolescents and tumor invasion, expressed by extension beyond the thyroid capsule and the presence of metastases in regional lymph nodes and lungs, was more prominent in the prepubertal than in the pubertal patients (8,9). Lazar et al (8) reported that DTC is more aggressive in prepubertal children with an increased incidence of extrathyroidal extension, lymph node involvement, and lung metastases at presentation compared with pubertal children.

In our cohort, in addition to the more disseminated presentation, recurrence rate was higher in the prepubertal group during follow-up. Some pediatric studies have shown that younger age is associated with persistent disease or recurrence. PTC with an onset at ages younger than 10 years appears to have higher recurrence and mortality rates than PTC with onset at older ages (7). In a study from Belarus on 740 children, younger age was related to an increased risk of recurrent lymph node and lung metastases after correcting for other risk factors (10). However, some studies have not confirmed these results (11,12). Lazar et al (8) reported that despite the aggressive presentation of DTC in prepubertal children, its course and outcome were similar to that of the pubertal group.

It has been advanced that PTC is a distinct disease not only in children and adults but also in prepubertal and pubertal children (7,8,9,10). BRAF^V600E^ mutation in pediatric PTC might be important to explain the difference encountered among prepubertal, pubertal children, and adults. In our cohort, although frequency of BRAF^V600E^ mutation is not so high, we did not find any association of BRAF^V600E^ mutation with lymph node or lung metastases or extrathyroidal involvement, findings consistent with most pediatric reports (19,20,24,25). While some adult studies have shown more aggressive clinical behavior and worse prognosis for patients with BRAF^V600E^ mutation, others have not (15,17,26,27). Meta-analyses in adults report a significant association of BRAF^V600E^ with lymph node metastases, tumor size, and extrathyroidal extension (17,27). BRAF^V600E^ mutation is a criterion for tumor aggressiveness in papillary microcarcinoma in some adult studies (28,29). However, Gouveia et al (26) demonstrated in a study of 429 adult patients with PTC that while 73.2% of patients had the BRAF^V600E^ mutation, there was no association with BRAF^V600E^ mutation and lymphovascular invasion, or extrathyroidal involvement. The prognostic value of BRAF^V600E^ mutation is not clear in pediatric patients. Henke et al (24) reported a high BRAF^V600E^ mutation rate (63%) in a pediatric population, but they did not find any correlation with BRAF^V600E^ mutation and a more extensive or aggressive disease. Some other pediatric studies also reported no association between BRAF^V600E^ mutation and the presence of an extensive disease (20,25,30). A need for large pediatric cohort studies is obvious.

Recent studies have shown that BRAF^V600E^ mutations are common in adult PTC patients (29%–83%) (31,32). The prevalence of BRAF^V600E^ mutation is variable in children ranging from 0% to 63% (19,24). We found BRAF^V600E^ mutation in 25% of our PTC patients. We were not able to show any correlation of BRAF^V600E^ mutation with age and pubertal status. There are controversial results on the correlation of BRAF mutation and age in the pediatric literature. Although it was reported that BRAF mutation increases with increasing age, our results are in line with other studies which demonstrated no correlation between BRAF and age (24,25,33).

The BRAF^V600E^ mutation is most commonly found in classical PTC in adults (17,26,33,34). As in adult patients, BRAF^V600E^ mutation in pediatric patients occurs more commonly in classical PTC than in non-classical subtypes (24,25). We also observed that BRAF^V600E^ mutation occurred mostly in classical PTC patients. In our group, there was no sex difference with respect to BRAF^V600E^ mutation. Henke et al (24) showed a male predominance in pediatric patients consistent with the findings on an adult population (17,35).

We found increasing numbers of cases of PTC diagnosed in the last 10 years at our unit after excluding patients with a history of radiotherapy. The reason for this increase in rate of PTC in pediatric patients, especially in the pubertal group, could be related to improved scrutiny for early diagnosis in recent years, since in our cohort 27.5% of the patients were diagnosed to have microcarcinoma within the last decade. Similarly, several studies have reported dramatic increases over recent decades in incidence of thyroid cancer, predominantly small papillary carcinomas (36,37,38). It was proposed that sudden changes in thyroid cancer incidence have resulted from large scale thyroid gland surveillance in high risk populations, improved diagnostic technology (ultrasonography, computed tomography, magnetic resonance imaging), and increased access to health care services (37,38).

In conclusion, our findings indicate that PTC is more disseminated in prepubertal children with an increased incidence of lateral neck lymph node involvement and lung metastases at presentation as compared with pubertal children. Extensive surgical treatment and repeated RAI treatment are required in prepubertal children. BRAF^V600E^ mutation frequency was not high in our patients and was comparable to most other pediatric studies. We showed that BRAF^V600E^ mutation was not correlated with a more extensive or aggressive disease process. Genetic factors other than BRAF^V600E^ may also be involved in the expression of biological features and clinical behavior of PTC in prepubertal and pubertal children and adults.

## Figures and Tables

**Table 1:**
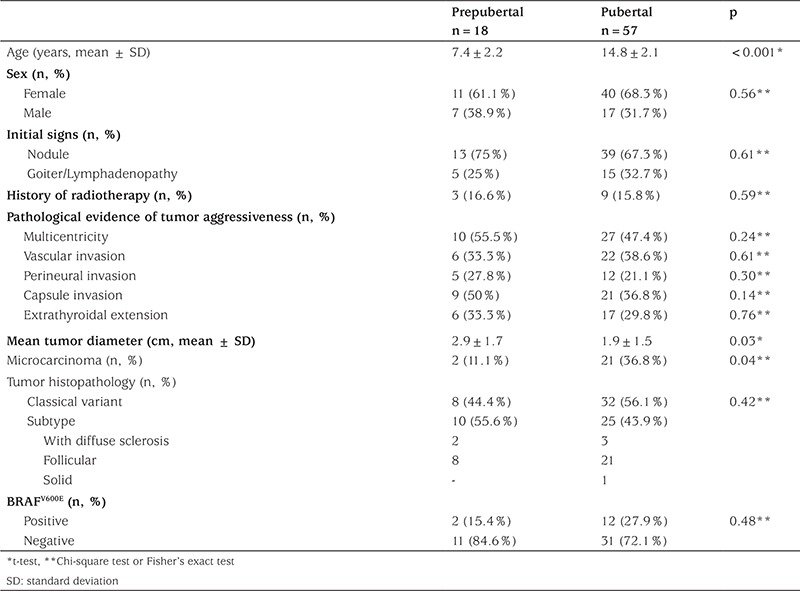
Comparison of some clinical and laboratory features of papillary thyroid cancer in prepubertal and pubertal patients

**Table 2:**
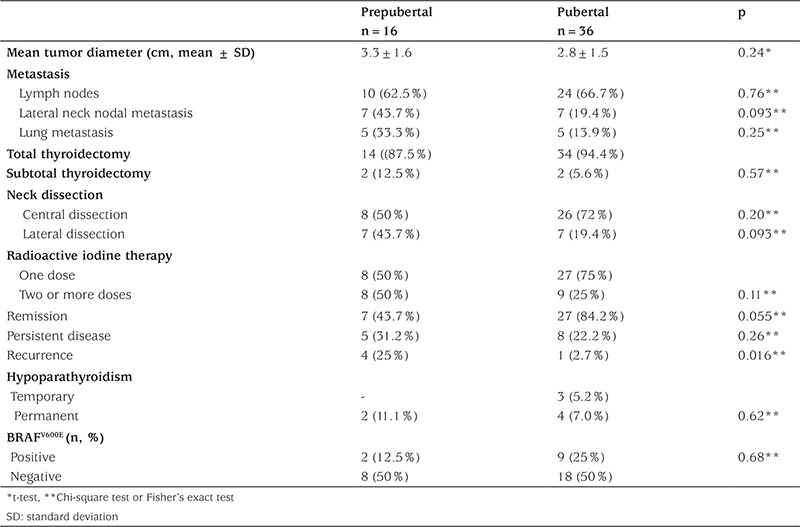
Comparison of type of treatment, outcome, and presence of BRAF^V600E^ mutation in prepubertal and pubertal patients after excluding microcarcinoma

**Table 3:**
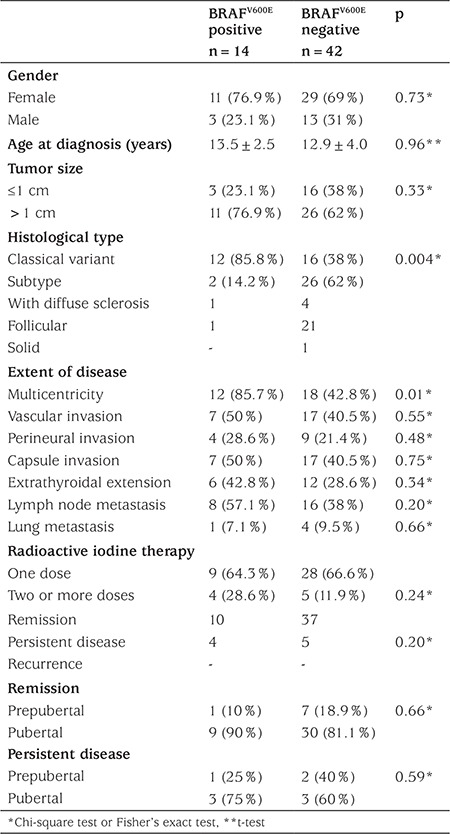
Comparison of some features of patients with respect to the presence of BRAF^V600E^ mutation

**Table 4:**
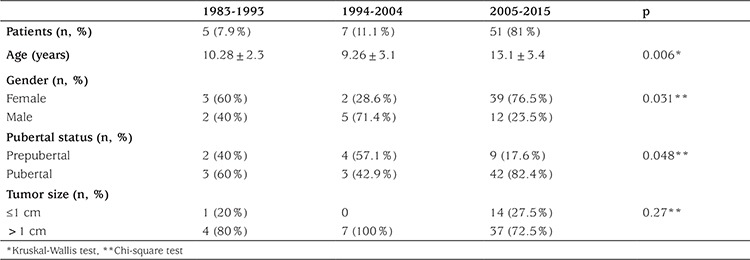
Number of patients with papillary thyroid cancer per every 10 years after excluding patients with a history of radiotherapy
